# Development and Validation of an Interpretable Artificial Intelligence Model to Predict 10-Year Prostate Cancer Mortality

**DOI:** 10.3390/cancers13123064

**Published:** 2021-06-19

**Authors:** Jean-Emmanuel Bibault, Steven Hancock, Mark K. Buyyounouski, Hilary Bagshaw, John T. Leppert, Joseph C. Liao, Lei Xing

**Affiliations:** 1 Laboratory of Artificial Intelligence in Medicine and Biomedical Physics, Stanford University School of Medicine, Stanford, CA 94304, USA; 2 Radiation Oncology Department, Hôpital Européen Georges Pompidou, Assistance Publique—Hôpitaux de Paris, 75015 Paris, France; 3 Department of Radiation Oncology, Stanford University School of Medicine, Stanford, CA 94305, USA; shancock@stanford.edu (S.H.); mbuyyou@stanford.edu (M.K.B.); hbagshaw@stanford.edu (H.B.); 4 Department of Urology, Stanford University School of Medicine, Stanford, CA 94305, USA; jleppert@stanford.edu (J.T.L.); jliao@stanford.edu (J.C.L.)

**Keywords:** prostate cancer, artificial intelligence, machine learning, prediction

## Abstract

**Simple Summary:**

This article presents a gradient-boosted model that can predict 10-year prostate cancer mortality with high accuracy. The model was developed and validated on prospective multicenter data from the PLCO trial. Using XGBoost and Shapley values, it provides interpretability to understand its prediction. It can be used online to provide predictions and support informed decision-making in PCa treatment.

**Abstract:**

Prostate cancer treatment strategies are guided by risk-stratification. This stratification can be difficult in some patients with known comorbidities. New models are needed to guide strategies and determine which patients are at risk of prostate cancer mortality. This article presents a gradient-boosting model to predict the risk of prostate cancer mortality within 10 years after a cancer diagnosis, and to provide an interpretable prediction. This work uses prospective data from the PLCO Cancer Screening and selected patients who were diagnosed with prostate cancer. During follow-up, 8776 patients were diagnosed with prostate cancer. The dataset was randomly split into a training (*n* = 7021) and testing (*n* = 1755) dataset. Accuracy was 0.98 (±0.01), and the area under the receiver operating characteristic was 0.80 (±0.04). This model can be used to support informed decision-making in prostate cancer treatment. AI interpretability provides a novel understanding of the predictions to the users.

## 1. Introduction

Each year in the United States, 180,000 patients are diagnosed with prostate cancer, and 26,120 men die from the disease. PSA testing has resulted in a significant increase in the diagnosis and treatment of prostate cancer [1,2]. But the management of low-risk prostate cancer management remains controversial [3]. Many men do not benefit from treatment because the disease is either indolent or disseminated at diagnosis. Every year, 35,000 men are being overdiagnosed with prostate cancer that will never cause symptoms or death and undergo unnecessary treatments causing complications because of screening [4].

The role of PSA-based screening in reducing mortality from prostate cancer is still controversial: The PLCO trial did not find any reduction in mortality [5,6,7]. On the other hand, the European Randomized Study of Screening for Prostate Cancer (ERSPC) study did find a substantial reduction in cancer-specific mortality: The overall relative risk of prostate cancer death was 0.46 (95% CI: 0.19–1.11) and 0.48 (95% CI: 0.17–1.36), in favor of screening [8]. For patients diagnosed with PCa, the ProtecT trial was conducted to compare the effectiveness of active monitoring, radical prostatectomy, and external-beam radiotherapy. Between 1999 and 2009, 82,429 men 50 to 68 years of age were tested with PSA, 2664 were diagnosed with localized PCa, and 1664 were randomized in three arms. At 10 years of follow-up, prostate-cancer-specific mortality was low, irrespective of the treatment (or absence of treatment) [8]. At 10 years, prostate-cancer-specific survival rates were 98.8% (97.4–99.5), 99% (97.2–99.6), and 99.6% (98.4–99.9) for active monitoring, surgery, and radiotherapy, respectively. Patients in the active monitoring group developed more metastases (*p* = 0.004) and had a higher rate of disease-progression (*p* < 0.001). Patients from the surgery and radiotherapy groups had significant sexual, urinary, and bowel function impairment from treatment [9].

In order to assess whether a patient with prostate cancer would benefit from cancer treatment, we created a model to predict the risk of death from prostate cancer 10 years after diagnosis that would take into account the patient’s comorbidities and cancer’s features. A gradient-boosting model was trained on the prospective data of the PLCO trial to provide a prediction and a visualization of the features explaining the outcome. We deployed the model in a web interface that can be used to obtain a personalized prediction and explanation in a format that can be readily implemented in a clinical setting.

## 2. Materials and Methods

### 2.1. Data

This model was trained on data from the prospective randomized multicenter trial PLCO where 76,693 men at 10 U.S. study centers were randomly assigned to receive either annual screening (*n* = 38,343) or usual care as the control (*n* = 38,350). A data transfer agreement was obtained from the National Cancer Institute (NCI), and the data was downloaded from the internet [10].

The dataset contains nearly all the PLCO study data available for prostate cancer screening, incidence, and mortality analyses. The dataset contains one record for each of the participants in the PLCO trial. The main package includes a comprehensive description of the patients included in the trial, as well as their complete follow-up. Patients that were diagnosed with prostate cancer during follow-up were selected to train and test the model, no matter in which arm of the trial they were included. Before any analysis, the dataset was split into a training and a testing dataset using an 80/20 ratio.

### 2.2. Feature Selection

Several tools, mainly nomograms, are currently available to predict relapse after surgery [11], or mortality [12,13,14,15,16]. We used features from the dataset relevant to prostate cancer diagnosis, medical history, physical activity, and socio-economic status of patients. The features included in the analysis were:

(1) Prostate cancer: PSA, T, N, M stage, Gleason score, and initial treatment (if performed)
(2) Medical history: Age, height, weight, current smoking status, smoking pack-years, daily alcohol consumption, history of prostatitis, nocturia, arthritis, bronchitis, diabetes, emphysema, heart attack, hypertension, liver disease, osteoporosis, stroke, elevated cholesterol.
(3) Physical activity: Activity at least once a month during the last year, physical activity at work
(4) Socio-economic status: Family income, education
(5) Hormonal status: Hair pattern at age 45, weight gain pattern

### 2.3. Predictions

A gradient-boosting machine model was trained to predict PCa mortality at 10 years with decision-tree-based learners using the XGBoost python package [17]. Survivals were calculated from the time of diagnosis. XGBoost is currently considered as the state-of-the-art for prediction with tabular data. XGBoost inherently handled missing values. [18]. Hyperparameters were selected on the training dataset, with a nested, cross-validation, with the Python package BayesianOptimization [19]. This approach provides a fast and efficient search of the optimal hyperparameters through Bayesian inference and Gaussian process, attempting to find the maximum value of an unknown function in as few iterations as possible. The class imbalance was compensated with the scale_pos_weight parameter of XGBoost.

To assess the performances of the models, we used the non-parametric bootstrap procedure: From the test dataset, we sampled all patients with replacement and evaluated the models on this sample. By repeating this process 200 times, we obtained a distribution of the performance metric and reported the 2.5 and 97.5 percentiles as 95 confidence intervals (CI).

A TRIPOD checklist is provided in File S1.

### 2.4. Model Interpretability

Understanding the predictions of the models is very important in our setting, because we need to know whether the prediction relies on the aggressivity of the PCa or on any other comorbidities of the patient. Shapley values [20] used the SHAP Python package to interpret the predictions [21]. We obtained the top 20 contributing features with an interpretation of how they participate in the prediction. At an individual scale, we can also visualize for any patient from the dataset, or any new patient, the features that participated in the prediction and how they influenced the final outcome.

### 2.5. Online Model Deployment

After training, the model was saved in a file that can be loaded. A web application was deployed online to perform new predictions with the Dash Python framework. Answers to thirty questions will provide the risk of dying from prostate cancer within 10 years from diagnosis.

## 3. Results

### 3.1. Cohort Description

In the PLCO trial, 8776 patients were diagnosed with prostate cancer (4579 patients were in the screening arm, and 4197 in the control arm). The dataset was split into two (7021 patients to train and 1755 to test the model). In total, 685 patients (6.2%) died from prostate cancer during follow-up. Patients’ characteristics are presented in Table 1.

### 3.2. Model Performances

Model performance was excellent with an accuracy of 0.98 (±0.01). The complete metrics are presented in Table 2.

**Figure 1:**
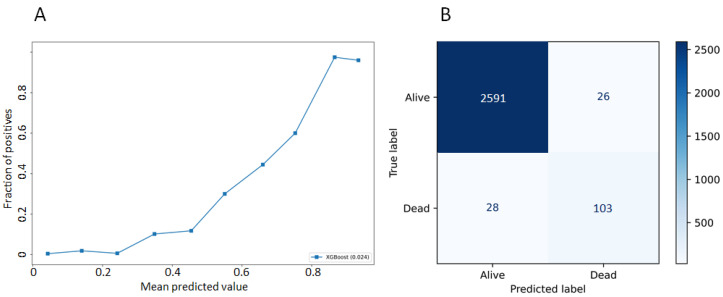
Calibration plot (**A**) and confusion matrix (**B**).

The model showed good calibration with a Brier score of 0.024. The calibration curve shows the predicted probabilities to die from prostate cancer within 10 years of diagnosis. We also provide a confusion matrix (Figure 1).

### 3.3. Most Important Features Explaining the Prediction

**Figure 2:**
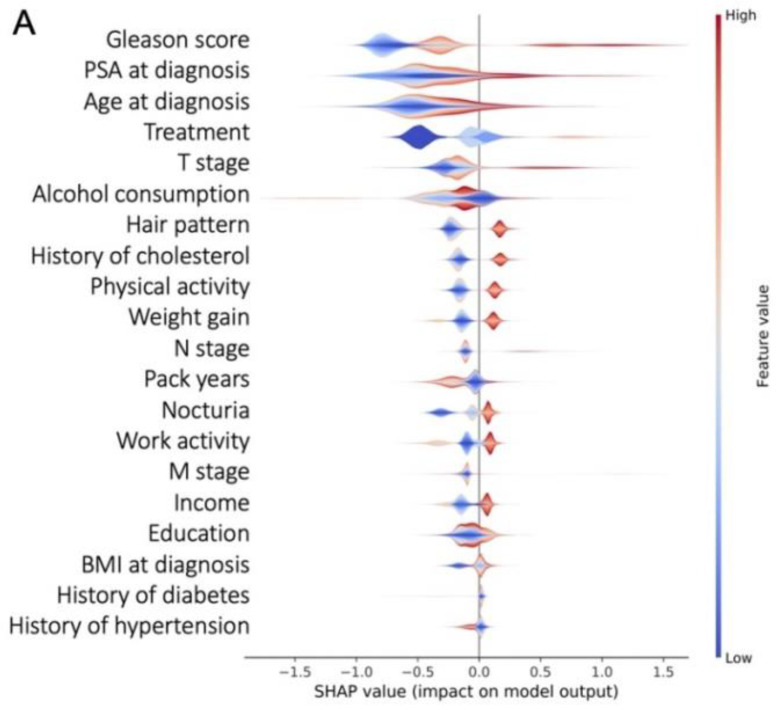

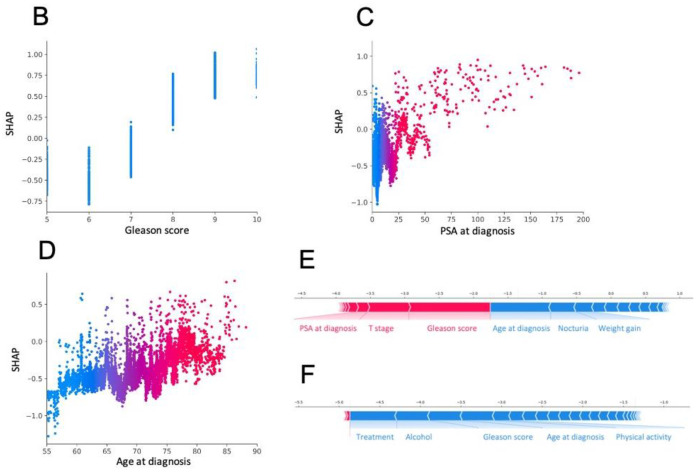
(**A**) The 20 most important features for prostate cancer mortality prediction. Population (**B**–**D**) and individual (**E**,**F**) level interpretability.

The five features that contributed most to model performance were the Gleason score, PSA at diagnosis, age, type of initial treatment, and T stage. Features related to general health status, such as alcohol consumption, hormonal status, and physical activity also, had a significant impact on the prediction (Figure 2A). Higher Gleason score, PSA levels, and age at diagnosis have a higher Shapley value, associated with a greater risk of death from PCa (Figure 2B–D). Each feature’s contribution is displayed on the x-axis. A feature with a negative Shapley value will decrease the risk of dying. The y-axis shows that a positive Shapley value increases the risk of dying and a low Shapley value decreases the risk of dying in Figure 2B–D. Higher Gleason score, PSA levels, and age at diagnosis have a higher Shapley value, associated with a greater risk of death from PCa.

Predictions for two types of patients: In Figure 2E, a high-risk prostate cancer (Gleason 9 (4 + 5), PSA = 24 ng/mL and T3bstage), without significant comorbidities (56 y.o., no smoking, no alcohol, with physical activity). In Figure 2F, intermediate-risk prostate cancer (Gleason 7 (3 + 4), PSA = 11 ng/mL and T2cstage), with several comorbidities (73 y.o., smoker, 53 pack-years, alcohol consumption, with physical activity). The first patient has a 19% probability of dying from prostate cancer. The aggressiveness of prostate cancer (in red) explains this probability. They are in part compensated by good prognosis factors, such as age (in blue). The second patient has a 1% probability of dying from prostate cancer.

### 3.4. Model Deployment Online and Interpretation at the Individual Scale

The model is available online: https://prostatecancersurvival.stanford.edu (accessed on 18 June 2021). For reproducibility, we also made the model available as a pickle object in a GitHub repository: http://github.com/jebibault/ProstateCancerSurvival (accessed on 18 June 2021). The object can be loaded to perform new predictions.

## 4. Discussion

The treatment of prostate cancer is based on clinical states that range from low grade/volume localized, locally advanced, metastatic castrate-sensitive, to metastatic castrate-resistant disease. In oncology, clinical management decisions are based on risk stratification, but defining high risk is a complex task in prostate cancer. Localized tumors include indolent diseases that are unlikely to result in morbidity or reduce life expectancy if left untreated, diseases curable with a single definitive modality, or cancer destined to relapse locally or systemically and result in death. This last category is currently considered as “high-risk”, but no classification scheme exists to provide a faithful prediction of the patients’ outcomes and consistently optimize therapeutic management. Even if it did exist, some high-risk patients would still die from competing comorbidities, before cancer could actually become fatal. A comprehensive algorithm that takes into account prostate cancer features, but also a more thorough description of the health status of a patient was needed to guide decision-making. This work has several clinical implications. It provides an accurate prediction of the mortality risk from prostate cancer 10 years after diagnosis. Being able to predict which patient is at risk of dying within 10 years will allow for informed discussion on the risk-benefit analysis of prostate cancer treatment can have a potential benefit for the patient. In this cohort from the PLCO trial, with 13 years of follow-up, only 499 patients (5.7%) died from PCa, while 2629 patients (30%) died from other causes. ProTecT confirmed in a prospective trial that all treatments had significant side-effects that negatively impacted the quality of life (QoL). If a patient will not benefit from PCa treatment because of other life-threatening conditions, the role of a physician should be to dissuade him from even getting treated for PCa, to prevent a deterioration of his QoL. In order to do so, being able to accurately predict the risk of dying at a reasonable time horizon is not sufficient. The algorithm needs to be interpretable to understand the features on which the prediction is made. A “black-box” model would only create more anxiety and would likely not provide actionable information. If the model didn’t explain why it’s making a prediction, we will not understand if a patient is at risk of dying because of the aggressive features of PCa, even if it is treated, or because of his comorbidities. Our model addresses this issue and provides detailed information by ranking features by importance, on an individual scale. In the literature, several nomograms and algorithms are currently available: Preoperative nomograms typically include factors, such as PSA level at diagnosis, clinical stage, and Gleason score [12,13]. Postoperative nomograms include these factors with surgical margins, capsular and seminal vesicle invasion, and regional lymph node status [11]. Some are available online [22], and most have been created with the data from a single center, which could potentially introduce significant biases and limit their generalizability.

Artificial intelligence (AI) techniques can be used in that setting [23]. The applications of AI are vast [24], and include diagnostic imaging [25], pathology [26], segmentation [27], prognostics [28], and automatic treatment planning [29,30]. Our model is the first to be trained on a large, prospective, and multicenter cohort of patients. Comparing the results from our model to results from other models is unfortunately currently not possible, because the models are currently not available for external validation. In that regard, we chose to release our model as a pickle object in a GitHub repository for replication or comparison studies. Because other datasets are not readily available, or because they do not include all the variables available in the PLCO dataset that we used to train our model, we cannot validate our results on an external cohort.

This study has several limitations: First, the PCLO trial was about prostate cancer screening and early detection and its relevance for prostate cancer overall survival. Some of the patients did have, and others were not supposed to have had early detection. Early detection and subsequent treatment might have had an impact on PCa death. The PCLO trial is also criticized because of its “PSA contamination” in patients without PSA screening. A significant portion of patients in the no screening arm had a PAS screening. Since we selected patients from both arms of the trial, this bias should be limited. But the prospective nature of the data and the fact that we selected patients from both arms of the trial, should limit biases. Another limitation is that the data mostly included patients that were diagnosed with localized prostate cancer, because only 195 patients (2.2%) were metastatic at diagnosis. This is because PLCO was a screening effectiveness trial and means that our model should probably be used with caution for these specific patients. As a self-assessed tool, the performance of the model could also be different, due to questionnaire response biases. But the fact that most of the data are from questionnaires that were given to patients during the trial could also limit this issue. Another issue we needed to address for modeling, was the inherent class imbalance of the dataset: This was considered during the training of the models by correcting the positive class. Finally, we need to consider how this model might be used in practice: The dilemma in this setting is obvious that patients in the PCLO trial died of PCA even though they were treated and hopefully received the best treatment possible. However, those in the trial who were successfully treated and consequently wer e saved from death, are not in the focus of our tool. Our tool mainly allows us to detect the patients who will die from causes that are different from PCa, and the patients who will die from PCa, even though they were treated. While the performance of the model is good for the whole cohort, there are significant challenges when calibrating the model to a single individual. The model should not be used as the only tool guiding a treatment strategy, but it could help guide decision making.

## 5. Conclusions

Using prospective data from the PLCO trial, we created a gradient-boosted model to predict the risk to die from prostate cancer 10 years after diagnosis with high accuracy, with only 30 clinical features, available at no cost. Because our model also provides interpretability, the prediction could be used to personalize treatments better.

## Figures and Tables

**Table 1:** Characteristics of patients with prostate cancer and patients who died from prostate cancer within 10 years after diagnosis in the PLCO trial.

Characteristic	No. (%) All Patients	No. (%) Patients Who Died from PCa
Age		
Under 65 years old	1990 (22.7)	109 (15.9)
Between 65 and 75 years old	5181 (59)	283 (41.3)
Over 75 years old	1605 (18.3)	293 (42.8)
Prostate Cancer		
Localized PCa	7668 (87.4)	436 (63.6)
Low-risk	2940 (33.5)	199 (29.1)
Intermediate-risk	3476 (39.6)	105 (15.3)
High risk	1252 (14.3)	132 (19.3)
Locally advanced PCa	913 (10.4)	122 (17.8)
Metastatic PCa	195 (2.2)	127 (18.5)
PSA		
<10 ng/mL	6516 (74.2)	254 (37.1)
10–20 ng/mL	1137 (13)	94 (13.7)
>20 ng/ml	1123 (12.8)	337 (49.2)
Gleason score		
Gleason ≤ 6	4744 (54.1)	353 (51.5)
Gleason 7	2842 (32.4)	158 (23.1)
Gleason 8	607 (6.9)	95 (13.9
Gleason ≤ 9	455 (5.2)	48 (7)
N/A	128 (1.5)	31 (4.5)
Treatment		
Surgery	3212 (36.6)	114 (16.6)
Radiotherapy	3607 (41.1)	201 (29.3)
Chemotherapy	1067 (12.2)	54 (7.9)
Hormonotherapy	654 (7.5)	161 (23.5)
N/A	236 (2.7)	155 (22.6)

**Table 2:** Performances of the survival model evaluated with the bootstrap method on the test dataset.

Metric	Definition	Result
Accuracy	Number of correct predictions/total number of input samples	0.98 (±0.01)
Precision	Number of correct positive predictions/number of positive predictions	0.80 (±0.1)
Recall	Number of correct positive predictions/number of all positive samples	0.60 (±0.08)
f1-score	Harmonic mean of the precision and the recall	0.66 (±0.07)
auROC	Area under the curve of the true positive rate and false positive rate at various thresholds	0.80 (±0.04)
prAUC	Area under the curve of precision and recall at various thresholds	0.54 (±0.07)

## Data Availability

Data are available from The Cancer Data Access System: https://cdas.cancer.gov/plco/ (accessed on 18 June 2021).

## Supplementary Materials

The following are available online at https://www.mdpi.com/article/10.3390/cancers13123064/s1, File S1: Tripod statement.
